# The efficacy of mifepristone combined with methotrexate for the treatment of ectopic pregnancy: a systematic review and meta-analysis[Author-notes FN0001]*

**DOI:** 10.1080/07853890.2022.2136747

**Published:** 2022-11-16

**Authors:** Qiling Su, Huiyan Feng, Tian Tian, Xiaoqian Liao, Yunhui Li, Xiaomao Li

**Affiliations:** Gynecology Laboratory, Department of Gynecology, The Third Affiliated Hospital of Sun Yat-Sen University, Guangzhou, China

**Keywords:** Ectopic pregnancy, meta-analysis, methotrexate, mifepristone, randomized controlled trials

## Abstract

**Objective:**

Systematically evaluate the clinical efficacy of mifepristone combined with methotrexate therapy for ectopic pregnancy (EP), analyze the experimental designs, put forward improvement ideas.

**Methods:**

RCTs of mifepristone combined with mifepristone for EP until January 2022 in six databases were searched. The primary outcome indicator was the cure rate. RevMan 5.4 was used to analyse and the online GRADEpro tool was used to assess the certainty of the evidence.

**Results:**

Twenty-five RCTs involved 2263 patients. The cure rate was higher in the investigational group (OR = 4.09, 95%CI: [3.20, 5.22]), time of vagina stopped bleeding (MD = −11.21, 95%CI: [−11.85, −10.57]) and time of abdominal pain disappeared (MD = −6.24, 95%CI: [−6.63, −5.86]) were shorter in the investigational group, β-HCG level (MD = −585.32, 95%CI: [−609.62, −561.03]) was lower and diameter of the mass (MD = −1.23, 95%CI: [−1.40, −106]) was smaller in the investigational group. The certainty of the evidence for most outcomes was moderate or high, and only one was low.

**Conclusions:**

The combination of mifepristone and methotrexate can improve the efficacy of ectopic pregnancy without amplifying the toxic side effects. Larger scale and better design of the randomized controlled trials are needed.

**KEY MESSAGES**
- In recent years, the increase in ectopic pregnancies and their impacts on female fertility makes physicians have to find an effective medical treatment as soon as possible that can avoid surgery.
- The mifepristone combined with methotrexate therapy for EP has better curative effects on improving the cure rate, lowering β-HCG level, reducing the mass, and alleviating symptoms of abdominal pain and bleeding, without amplifying the toxic side effects.
- Literature with high quality is lacking, and well-designed, large-scale and high-quality multicenter randomized controlled trials are needed.

## 1. Introduction

Ectopic pregnancy (EP) is when a blastocyst is implanted outside the uterus, and most are fallopian tube pregnancies. EP is a common clinical acute abdominal disease in obstetrics and gynaecology, as well as one of the primary causes of maternal death. In recent years, the incidence of EP has increased year by year and has shown a trend of patients of younger age, which makes it important to effectively save patients’ lives and furthest preserve their fallopian tube function and fertility. literature reports that the minimum age of onset of EP is 16 years old [1], the incidence rate in pregnant women is 2% while the mortality rate accounts for about 10% of the total maternal deaths [2]. In Liu Ying’s study, the incidence rate increased from 2.48% in 2005 to 4.36% in 2012, and the proportion of conservative treatment increased from 7.14% to 20.73% [3]. The proportion under 20 years old in the later group increased from 1.1% 10 years ago to 4.5%. Unmarried women increased from 21.5% to 41.4%; The number of childless women rose from 45.7% to 66.8% [4]. Therefore, patients with EP tend to be younger, unmarried and childless.

Meanwhile, because of the various diagnostic methods, the widespread hygienic knowledge, and the wide application of transvaginal ultrasound examination and β-HCG test, it is possible to early diagnose EP and create opportunities for conservative treatment [5]. Since embryo implantation in the uterus is a complex developmental process, the molecular interaction mechanism is still unclear, especially for tubal EP. What is certain is that once pregnant, the trophoblast cells begin to secrete a large amount of HCG, and at the same time, the trophoblast cells also secrete substances that can distinguish between normal pregnancy and abnormal pregnancy [4]. Research has shown that the β-HCG level of EP patients is lower than that of normal pregnant women because the blastocyst cannot receive enough blood supply, the development of the placenta is limited and the trophoblast layer is mostly dysplastic [6]. Therefore, the β-HCG level can be used as an index of diagnosis and differential diagnosis of EP. On the other hand, as the disease progresses, the gestational sac (mass) will grow, and the patient may experience sudden and intense abdominal pain, irregular vaginal bleeding and other symptoms. The clinical symptoms above can be detected by ultrasound examination, which can also be considered an appropriate tool for the diagnosis of EP.

Drug therapy is one of the conservative treatments for EP. It is confirmed that drug therapy can prevent patients from surgical trauma and complications, for example, pelvic adhesion and furthest preserve fallopian tube function and increase fertility rate [7]. Nowadays, the common drugs include methotrexate, mifepristone, hypertonic glucose, prostaglandin, potassium chloride, fluorouracil, traditional Chinese medicine, etc., among which methotrexate and mifepristone are the most widely used.

Mifepristone is a steroidal anti-progesterone drug, the main principle of its treatment of EP is: blocking the secretion of progesterone to shrink corpus luteum, causing cell degeneration, decidua and chorion decrease. Mifepristone can also promote the release of endogenous prostaglandins, causing uterine contractions, cervical softening and dilation, and assisting ectopic embryo tissue discharge [8].

Methotrexate is an anti-tumor drug, mainly anti-folate, it binds effectively with folate reductase pharmacologically, and strongly inhibits dihydrofolate reductase in a competitive way, so that dihydrofolate cannot be transformed into tetrahydrofolate, which acts as a coenzyme in the synthesis of purine nucleotides and thymine nucleotides, thus destroying the synthesis of DNA, and competitively inhibiting the effective synthesis of DNA, RNA and other proteins. MTX can also lead to embryonic cell necrosis by inhibiting the production of embryonic nourishing cells, and it has a good curative effect in early treatment. Gestational trophoblastic cells have high sensitivity to methotrexate and they cannot continue to grow after EP patients take MTX, and the embryo will stop developing, eventually leading to necrosis, shedding of the embryo, which would gradually be absorbed [9,10].

In recent years, there have been many studies on mifepristone combined with methotrexate therapy [11], some of them showed that the combination therapy can dissolve trophoblast cells more quickly than methotrexate therapy, so the combination therapy has a lower risk of salpingorrhexis or peritoneal hemorrhage [12]. A number of studies have shown that the curative effect of combination therapy is better than mifepristone therapy and methotrexate therapy, while the incidence rates of the three therapies are not significantly different [13]. The mechanism of the combination therapy on EP is still unclear. It is speculated that the effect of methotrexate on the trophoblast may enhance the anti-decidual effect of mifepristone, leading to the destruction of the cervical trophoblast [14]. Some researchers believe that this is because the mechanisms and therapeutic targets of two drugs treating EP are different, so their combination can play a synergism and enhance their curative effect. Studies have shown that mifepristone combined with methotrexate is effective in the treatment of cervical pregnancy and interstitial pregnancy [15]. Probably the effect of Mifepristone on decidua is the reason for his efficacy in the treatment of intrauterine pregnancy or interstitial pregnancy [16].

Although most researchers come to the conclusion that the curative effect of combination therapy is better than that of monotherapy, other researchers believe that the difference between their efficacy is not significant, which is due to the poor quality of most trials, while combination therapy brings more adverse reactions [17]. According to Rozenberg’s article in 2003 [17], although many pieces of research have concluded that the efficacy of combination therapy was better than that of mifepristone therapy, they had the following defects limiting their interpretation of the research results: (1) their trials were not double-blind trials; (2) the size of a small sample (25 per arm) was not based on the pre-specified calculation; (3) The random group method was not clearly stated. Therefore, no accurate conclusion was drawn in the world, and opinions on which therapy is more effective vary.

On the other side, there are few RCTs about the combination therapy on EP, while there are lots of research with uneven quality and different methods. There was no related meta-analysis article in recent years and only one meta-analysis article in 2011 which was lack of flow charts, basic information and forest maps and has not been registered on Prospero.

Thus, on the one hand, this study will analyze the efficacy and come to a conclusion, based on the existing research. In this regard, we suspect that due to the different mechanisms of mifepristone and methotrexate on EP, their curative effect on EP can be amplified by superposition, without amplification of toxic and side effects, which is more conducive to maternal expulsion of foreign bodies. On the other hand, this study aims at filling the gap of relevant analysis, evaluate the advantages and disadvantages of inclusion trials, find out the reasons why most of the research results are not reliable enough, and put forward improvement measures and scientific designing schemes.

## 2. Data and methods

### 2.1. Inclusion criteria for study selection

#### 2.1.1. Types of studies

PRISMA guideline was followed. Clinical randomized controlled trials (RCTs) containing mifepristone combined with methotrexate therapy for EP were included, with no limitation of language and publication status.

#### 2.1.2. Types of participants

According to clear and recognized diagnostic criteria and efficacy criteria, all patients were diagnosed as EP, with stable vital signs, without broken mass or signs of active bleeding, without liver or kidney dysfunction or hematological system diseases, and size of the mass in ultrasound examination, as well as β-HCG level, met the conditions of conservative treatment, and the consent of patients and their families was obtained regardless of age, marital status, gestational age and source of the cases.

#### 2.1.3. Types of interventions

The experimental intervention is mifepristone combined with methotrexate therapy, without any other therapies. The control intervention is only mifepristone therapy, without any other therapies.

#### 2.1.4. Types of outcome measures

The clinical cure rate will be used as a primary outcome indicator, with the criterion of recovery clearly defined. After literature inclusion by primary outcome indicator, all of the remaining outcome measures will be used as secondary outcome indicators, regardless of type.

### 2.2. Exclusion criteria

Non-randomized controlled trials, including cross-over trials; Participants are scar pregnancy patients or cervical pregnancy patients; Using Chinese medicine therapy, operation therapy or other therapies as treatment in either group; Using different dosage or drug delivers between groups; More than one control group; Without cure rate as an outcome indicator.

### 2.3. Retrieval methods and strategies

We searched six electronic databases, PubMed, Cochrane Library, Embase, the China National Knowledge Infrastructure (CNKI), Wanfang Database (WF) and the Chinese Science and Technology Periodical Database (VIP) were included. RCTs published up to January 2022 were selected, without any language or publication restriction. The search strategy is shown in Table 1. Non-randomized controlled trials including meeting abstracts will be excluded by manual searching.

**Table 1.** Retrieval strategies.

ID	Query
#1	‘Pregnancy, Ectopic’[Mesh]
#2	(((((((Ectopic Pregnancies[Title/Abstract])OR(Pregnancies,Ectopic[Title/Abstract]))OR
	(Pregnancy,Extrauterine[Title/Abstract]))OR(Extrauterine Pregnancies[Title/Abstract]))OR
	(Extrauterine Pregnancy[Title/Abstract]))OR(Pregnancies,Extrauterine[Title/Abstract]))OR
	(Ectopic Pregnancy[Title/Abstract])))
#3	#1 OR #2
#4	‘Mifepristone’[Mesh]
#5	(((((((((((((((ZK98296[Title/Abstract])OR(ZK98296[Title/Abstract]))OR(ZK98296
	[Title/Abstract]))OR(R38486[Title/Abstract]))OR(R38486[Title/Abstract]))OR
	(RU486[Title/Abstract]))OR(RU486[Title/Abstract]))OR(RU486[Title/Abstract]))OR
	(R38486[Title/Abstract]))OR(RU38486[Title/Abstract]))OR(RU38486[Title/Abstract]))OR
	(RU38486[Title/Abstract]))OR(Mifegyne[Title/Abstract]))OR(Mifégyne[Title/Abstract]))OR
	(Mifeprex[Title/Abstract]))))
#6	#4 OR #5
#7	‘methotrexate’[Mesh]
#8	((((((((((((Amethopterin[Title/Abstract])OR(Methotrexate,(D)Isomer[Title/Abstract]))OR
	(Methotrexate,(DL)Isomer[Title/Abstract]))OR(Mexate[Title/Abstract]))OR
	(Methotrexate Sodium[Title/Abstract]))OR(Sodium,Methotrexate[Title/Abstract]))OR
	(Methotrexate,Sodium Salt[Title/Abstract]))OR(Methotrexate,Disodium Salt[Title/Abstract]))
	OR(Methotrexate Hydrate[Title/Abstract]))OR(Hydrate,Methotrexate[Title/Abstract]))OR
	(Methotrexate,Dicesium Salt[Title/Abstract]))OR(Dicesium Salt Methotrexate
	[Title/Abstract])))
#9	#7 OR #8
#10	#3 AND #6 AND #9

Ab: abstract; Mesh: medical subject headings; Ti: title.

### 2.4. Data extraction and management

#### 2.4.1. Literature inclusion and data extraction

We used RevMan5.4 to exclude the repetitive literature and then selected literature by reading the titles and abstracts, according to Inclusion and Exclusion Criteria. And we read the full text of the remaining literature to obtain the final inclusion literature.

Data were extracted according to the inclusion literature, which included general trial characteristics (authors and year), patient and disease data (age, gender, sample size and disease course), interventions (dosage and treatment course), control interventions and outcomes (outcome measures, adverse events, diagnostic criteria and baseline) and other clinical data (basic therapy and common therapy).

#### 2.4.2. Bias estimation

RevMan 5.4 was used for entry. The risk of bias in trials was assessed based on the following six items: random allocation, allocation concealment, blinding of participants, blinding of researchers, follow-up visits and complete cases and complete outcome measures. We categorized each item into one of three levels – ‘high risk’, ‘low risk’ or ‘unclear risk’ when 1 point for those at low risk and 0 points for those at high or unclear risk. Finally, we calculated the total points of each study and assess the total risk of each study as follows: 0–2 points are categorized as high risk, 3–4 points are categorized as unclear risk and 5–6 points are categorized as low risk.

#### 2.4.3. Outcome indicator

The cure rate is considered to be the primary indicator to evaluate the clinical curative effect, and other outcome measures of the included trials are used as the secondary indicator: β-HCG level, the time of β-HCG returned to normal, the time of vagina stopped bleeding, the time of abdominal pain’s disappearance, the time of disappearance of the mass, the diameter of the mass. The remaining outcome indicators are not analyzed because the number of their trials is less than two: pregnancy rate after treatment, repetitive dose rate, the proportion of mass reduction ≥30%, time of menstruation returned to normal, fallopian tube patency rate, the proportion of β-HCG level decreased ≥15%.

The cure rate is the percentage of the cured cases in the total cases. Patients are deemed to be cured when their clinical symptoms were alleviated or even disappeared, blood β-HCGs levels significantly decreased by more than 15% or even returned to normal, and pelvic mass was significantly reduced or even disappeared under ultrasound observation, and it is deemed to be ineffective when their clinical symptoms including recurrent abdominal pain were existing, blood β-HCGs levels were not decreased or even worsen, and the mass did not shrink or even increased.

### 2.5. Statistical analysis

#### 2.5.1. Quantitative data synthesis

Meta-analysis was performed by using RevMan5.4 software. Data for the cure rate is dichotomous, and others are continuous. Dichotomous data were expressed as the odds ratio (OR), and continuous data were expressed as mean difference (MD), both with a 95% confidence interval (95%CI).

#### 2.5.2. Assessment of heterogeneity

The heterogeneity test is carried out first among all studies, *I*² test and *Q*-test are used, and choose the analytic model according to the test. When *p* > 0.1 and *I*² < 50%, which means there is obvious heterogeneity, the fixed-effect model will be used; When *p* ≤ 0.1 and *I*² ≥ 50%, which means heterogeneity is not obvious, the random-effect model will be used.

#### 2.5.3. Publication bias

When the number of qualified RCTs reaches 10, we will use the inverted funnel plot to test the potential publication bias.

#### 2.5.4. Certainty of evidence

The certainty of the evidence for each outcome was assessed by using the Grading of Recommendations Assessment, Development and Evaluation criteria (GRADE) and approach to conduct management recommendations by the GRADEpro Guideline Development Tool (GDT) online (https://gradepro.org/).

## 3. Results

### 3.1. Trials description

**Figure 1.**
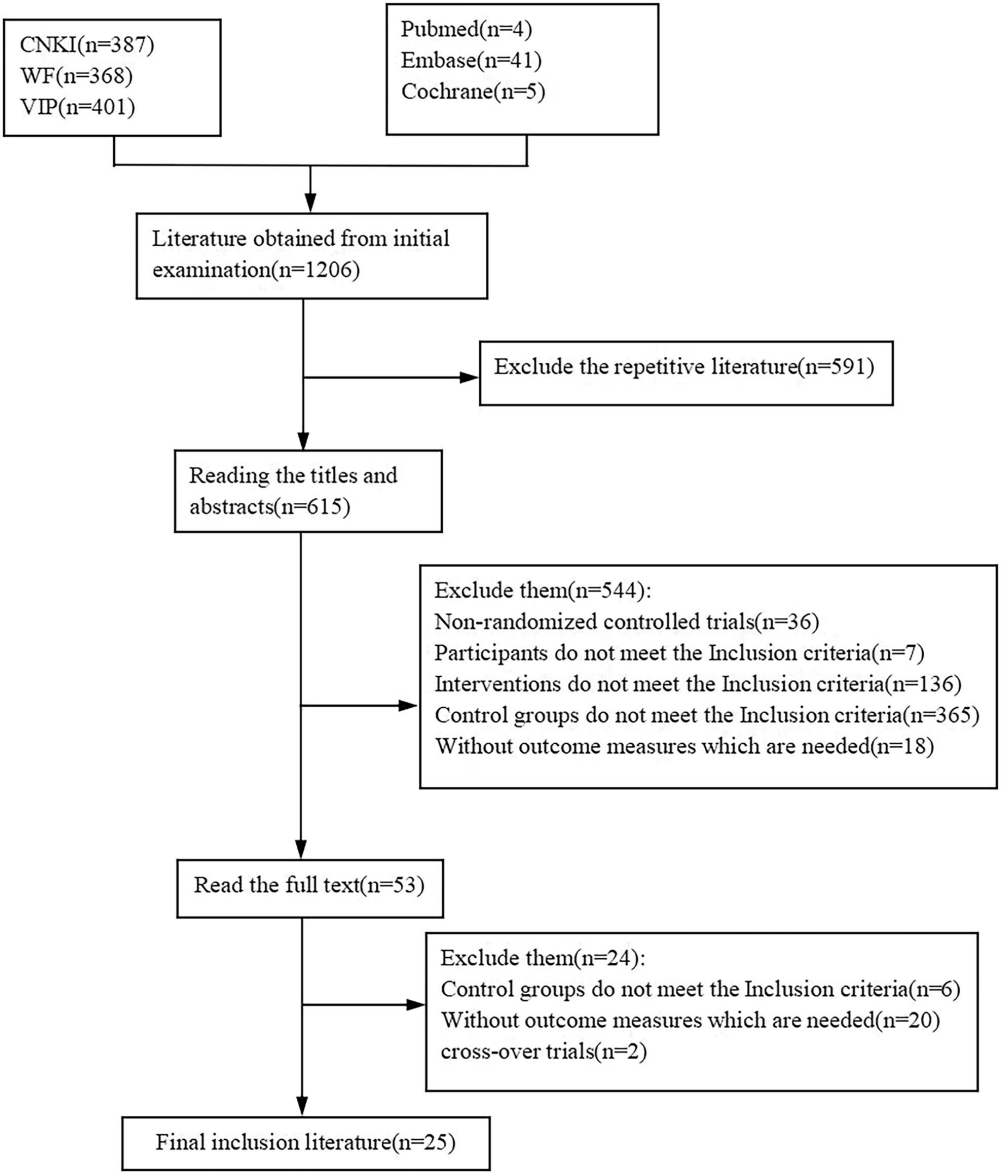
Literature selecting flow chart.

A total of 1206 trials (1156 trials in the Chinese database and 50 trials in the foreign language database) were initially searched from 6 databases, and 544 trials were excluded by reading titles and abstracts: non-RCTs (36), whose participants did not meet the inclusion criteria (7), interventions did not meet the Inclusion Criteria (136), control groups did not meet the Inclusion Criteria (365) and without outcome indicators which were needed (18). After reading the full text of the remaining 53 trials, 28 trial references were excluded due to the fact that their control groups did not meet the inclusion criteria or did not contain the required indicators. The inclusion process is shown in Figure 1.

### 3.2. Included studies

Twenty-five references were included in the final inclusion literature [18–42]. All trials were RCTs with two parallel arms, which were published up to January 2022. The general characteristics of the included trials are summarised in Table 2.

**Table 2.** Characteristics of the trials included in the meta-analysis.

Source	Sample T/C	Course (day)	Age (year)	Intervention		Treatment period (day)	Adverse reaction T/C	Outcome measure
				T	C			
Qixin Zhang [18]	46/46	T: 45～63	T: 28.5 ± 3.0	Mifepristone (25 mg, bid, 5 d) Methotrexate (1st day: 40 mg, after: 20 mg/d, im,5 d in total)	Mifepristone (25 mg, bid, 5 d)	5	3/7	a, b, c, d
		C: 48～60	C: 29.1 ± 3.2					
Yinjuan Huang [19]	50/42	T: 42 ∼ 56	T: 19 ∼ 43	Mifepristone (50 mg,bid,300 mg in total) Methotrexate (50 mg, im)	Mifepristone (50 mg, bid, 300 mg in total)	3	Gastrointestinal reaction	a, b, e, f
		C: 42 ∼ 56	C: 19 ∼ 43					
Changfeng Zhang [20]	52/52	/	T: 18 ∼ 46	Mifepristone (1st day:150 mg/d, 2nd, 3rd day: 75 mg, bid) Methotrexate (50 ms, im)	Mifepristone (1st day:150 mg/d, 2nd, 3rd day: 75 mg, bid)	3	/	a, b, c, g
			C: 18 ∼ 46					
Lianfen Bai [21]	42/42	T: 70 ∼ 84	T: 26.5 ± 3.2	Mifepristone (1st day: 150 mg/d, 2nd, 3rd day: 75 mg, bid) Methotrexate (50 ms, im)	Mifepristone (1st day:150 mg/d, 2nd, 3rd day: 75 mg, bid)	3	/	a, b, c, g
		C: 70 ∼ 84	C: 26.5 ± 3.3					
Lin Li [22]	50/50	T: 41.6 ± 3.6	T: 30.5 ± 2.4	Mifepristone (50 mg, bid) + Methotrexate (im, 50 mg, qd)	Mifepristone (50 mg, bid)	5	2/13	a, c, h, i, j
		C: 41.5 ± 4.2	C: 31.2 ± 3.3					
Haiying Lu [23]	46/46	/	T: 26.3 ± 3.1	Mifepristone(150 mg/d, qd, 3 d) Methotrexate (50 mg/m²,im)	Mifepristone (150 mg/d, qd, 3 d)	3	0/0	a
			C: 25.7 ± 2.4					
Cuilan Zhou [24]	51/49	T: 43.82 ± 6.25	T: 25.33 ± 4.67	Mifepristone (150 mg/d, qd, 3 d) Methotrexate (50 mg/m²,im)	Mifepristone (150 mg/d, qd, 3 d)	3	15/12	a
		C: 43.82 ± 6.25	C: 25.33 ± 4.67					
Ting Zhu [25]	42/42	T: 47.2 ± 5.7	T: 28.4 ± 3.2	Mifepristone (50 mg, bid, 5 d) Methotrexate (50 mg, im)	Mifepristone (50 mg, bid, 2 d)	5、2	14.29%/11.9%	a, b, c
		C: 46.9 ± 6.1	C: 27.9 ± 3.6					
Huiqiang Yu [26]	19/19	T: 41.7 ± 13.6	T: 27.5 ± 4.6	Mifepristone (1st day:150 mg/d, 2nd, 3rd day: 75 mg, bid) Methotrexate (50 mg/d, im)	Mifepristone (1st day:150 mg/d, 2nd, 3rd day: 75 mg, bid)	3、7	/	a, c, h, i, j
		C: 42.3 ± 12.9	C: 27.8 ± 4.5					
Xinyan Liu [27]	40/40	T: 40.2 ± 19.5	T: 28.6 ± 3.4	Mifepristone (100 mg, qd, 5 d) Methotrexate (20 mg, im, qd, 5 d)	Mifepristone (200 mg, qd, 5 d)	5	8/12	a, i, k
		C: 41.2 ± 18.9	C: 28.3 ± 3.6					
Yan Jiang [28]	15/15	T: 40.8 ± 14.3	T: 26.5 ± 4.3	Mifepristone (1st day:150 mg/d, 2nd, 3rd day: 75 mg, bid) Methotrexate (50 mg/d, im)	Mifepristone (1st day:150 mg/d, 2nd, 3rd day: 75 mg, bid)	3、7	/	a, c, h, i, j
		C: 41.4 ± 11.6	C: 25.1 ± 5.2					
Min Song [29]	50/50	T: 55.17 ± 4.12	T: 28.23 ± 2.64	Mifepristone (1st day:150 mg/d, 2nd, 3rd day: 75 mg, bid) Methotrexate (50 mg/d, im)	Mifepristone (1st day:150 mg/d, 2nd, 3rd day: 75 mg, bid)	3、7	/	a, c, h, i, j
		C: 52.41 ± 2.61	C: 31.23 ± 2.71					
Cuilian Feng [30]	19/19	T: 29.1 ± 3.7	T: 27.2 ± 5.4	Mifepristone (1st day:150 mg/d, 2nd, 3rd day: 75 mg, bid) Methotrexate (50 mg/d, im)	Mifepristone (1st day:150 mg/d, 2nd, 3rd day: 75 mg, bid)	3、7	5/5	a, i, k
		C: 28.6 ± 3.2	C: 28.4 ± 5.2					
Anxing Liu [31]	80/66	/	T: 29.4 ± 5.2	Mifepristone (administered at draught: 300 mg, after 24 h: 200 mg) Methotrexate (50 mg/m²+NS, 4 ml, im)	Mifepristone (administered at draught: 300 mg, after 24 h: 200 mg)	/	/	a, b, g, l, m
			C: 29.4 ± 5.2					
Yueping Deng [32]	55/55	T: 45.2 ± 3.1	T: 27.8 ± 3.2	Mifepristone (100 mg/d) Methotrexate (0.4 mg/kg d)	Mifepristone (100 mg/d)	5	8/5	a, i, k
		C: 45.2 ± 3.1	C: 27.8 ± 3.2					
Chunyan Zhang [33]	50/50	T: 51.3	T: 28.6	Mifepristone (50 mg, bid, 3 d) Methotrexate (50 mg/m², im)	Mifepristone (50 mg, bid, 3 d)	3	/	a, i, k
		C: 51.3	C: 28.6					
Rong Li [34]	58/58	T: 53.5 ± 2.3	T: 28.6 ± 4.5	Mifepristone (50 mg, bid, 3 d) Methotrexate (75 mg, im)	Mifepristone (50 mg, bid, 3 d)	3	/	a, i, k
		C: 53.5 ± 2.3	C: 28.6 ± 4.5					
Shizhen Zhong [35]	90/90	T: 55.4 ± 7.8	T: 27.8 ± 3.2	Mifepristone (50 mg, bid, 3 d) Methotrexate (50 mg/m²,im)	Mifepristone (50 mg, bid, 3 d)	3	8.9%/10.0%	a, b, g
		C: 56.0 ± 7.7	C: 27.9 ± 3.7					
Fang Liu [36]	60/60	T: 35 ∼ 68	T: 24	Mifepristone (100 ∼ 150 mg/d, 600 mg in total) Methotrexate (50 mg, im, 3 times)	Mifepristone (100 ∼ 150 mg/d, 600 mg in total)	3	10.0%/11.7%	a, b, g
		C: 35 ∼ 68	C: 24					
Guihua Liu [37]	48/48	T: 39.5 ± 18.3	T: 25.9 ± 4.1	Mifepristone (100 mg, bid) Methotrexate (20 mg, im, qd ,4 ∼ 5 times)	Mifepristone (100 mg, bid)	5	4/5	a, c, h, i, j
		C: 39.5 ± 18.5	C: 25.4 ± 4.2					
Zhimin Xu [38]	36/36	T: 45.61 ± 8.83	T: 27.52 ± 5.78	Mifepristone (25 ∼ 50 mg, bid) Methotrexate (0.4 mg/kg d, qd)	Mifepristone (25 ∼ 50 mg, bid)	15	4/3	a, b, c, g
		C: 45.56 ± 8.67	C: 27.43 ± 5.46					
Rui Chen [39]	56/53	T: 50.6 ± 4.9	T: 27.1 ± 1.8	Mifepristone (150 mg, qd) Methotrexate (50 mg/m², im, qd)	Mifepristone (150 mg, qd)	3	3/4	a, i, k
		C: 51.2 ± 5.8	C: 27.5 ± 2.4					
Qiulian Deng [40]	35/35	T: 45.5 ± 2.5	T: 29.5 ± 1.3	Mifepristone (50 mg, bid) Methotrexate (50 mg/d, im)	Mifepristone (1st day:150 mg administered at draught, 50 mg, bid)	4	0/0	a, i, k
		C: 43.2 ± 3.5	C: 28.7 ± 1.2					
Xuezhen Zhang [41]	32/32	T: 45.5 ± 3.5	T: 30.6 ± 3.8	Mifepristone (100 mg, bid) Methotrexate (80 mg, im, bid)	Mifepristone (100 mg, bid)	3	1/2	a, b, n
		C: 44.5 ± 3.5	C: 28.6 ± 3.2					
Hong Chen [42]	23/23	T: 31 ∼ 62	T: 26.3 ± 1.2	Mifepristone (100 mg, bid) Methotrexate (50 mg, im, qd)	Mifepristone (100 mg, bid)	/	2/3	a, c, i, j
		C: 31 ∼ 62	C: 26.3 ± 1.2					

I: investigational group; II: control group; /: not mention; a: cure rate; b: time of β-HCG returning to normal; c: time of disappearance of the mass; d: time of menstruation return to normal; e: pregnancy rate after treatment; f: repeat dosing rate; g: hospital stays; h: time of vagina to stop bleeding; i: β-HCG Level; j: time of disappearance of abdominal pain; k: diameter of the mass; l: ratio of β-HCG decreased by more than 15%; m: ratio of mass decreased by more than 30%; n: tubal patency rate.

### 3.3. Adverse events and adverse reactions

As is shown in Table 2, four trials showed that the incidence of adverse reactions in the investigational group was higher than that in the control group, while nine trials showed that the incidence of adverse reactions in the investigational group was lower than that in the control group. However, eight trials did not mention the existence of adverse events, two trials specified no adverse events, one trial mentioned the existence of gastrointestinal reaction but no significant differences between the two groups, and one trial showed the same incidence of adverse reactions between the two groups. All the relevant trials claimed that there was no significant difference between the two groups.

### 3.4. Risk of bias in the included trials

**Figure 2.**
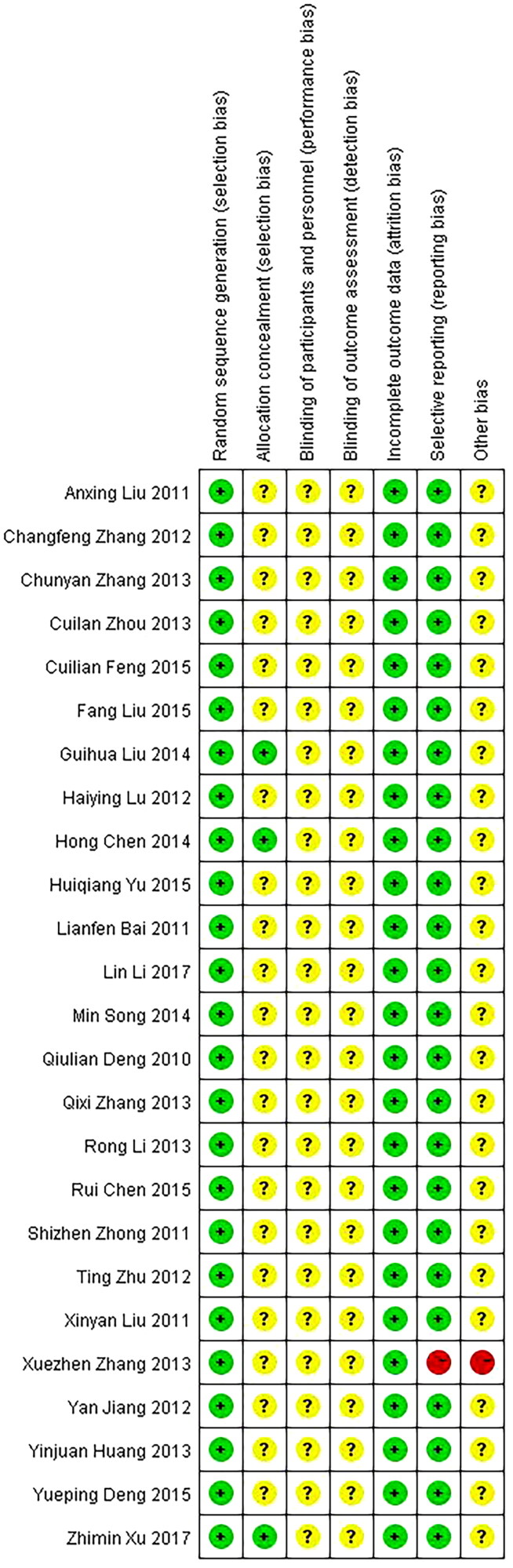
Risk of bias summary.

**Figure 3.**
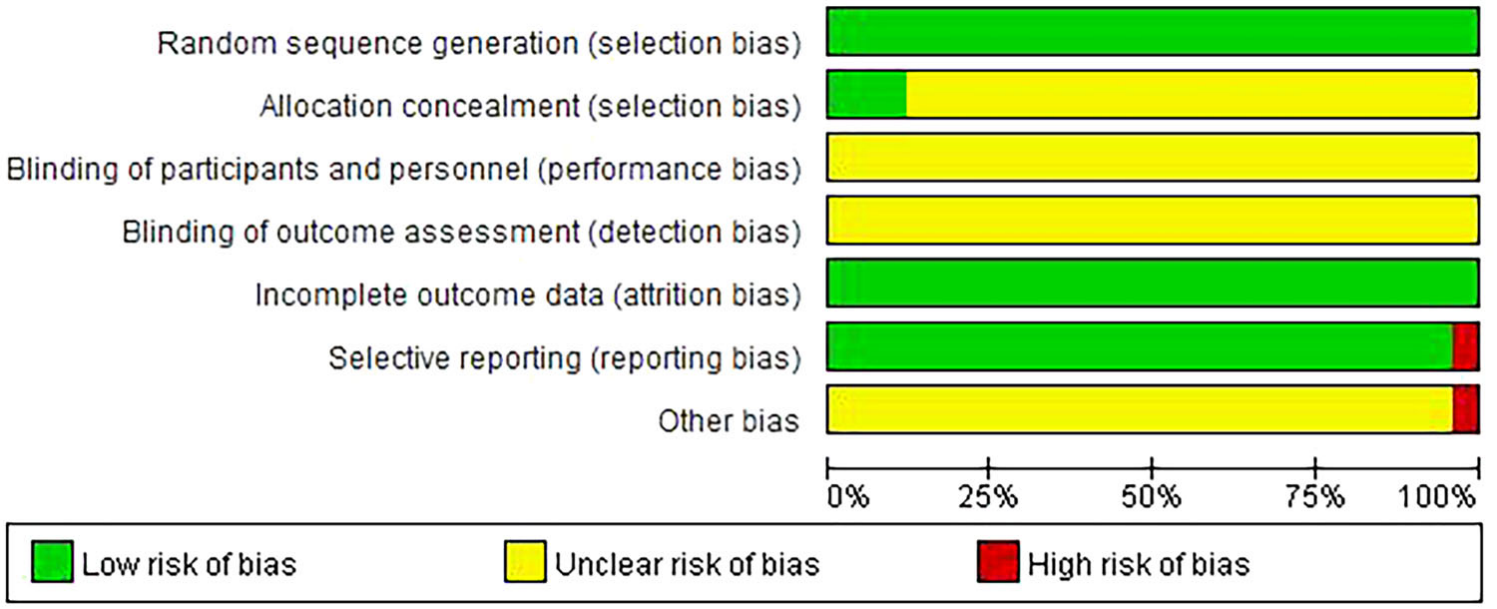
Risk of bias graph.

The quality assessment of the included trials is shown in Table 3. All trials used the randomized grouping method, and three of them used the random number table [37,38,42], one trial used random odd-even number grouping [35] and other trials did not mention using which random grouping methods. Moreover, all the trials that used the random number table had allocation concealment, and other trials did not mention it. None of the trials mentioned the double-blind, and cases of all trials had data integrity. Outcome indicators data of most trials are complete except for one trial [41], due to the fact that a few cases in this trial were lost in the follow-up of tubal patency. Thus it can be seen, most trials were generally low in quality and contained risks of bias, suggesting that future studies might influence the confidence intervals in the meta-analysis, and require revising the conclusion. The risk of bias graph and summary are shown in Figures 2 and 3.

**Table 3.** Quality assessment of the trials included in the meta-analysis.

Study	Random sequence generation (selection bias)	Allocation concealment (selection bias)	Blinding of personnel	Blinding of patients	Follow-up/ integrity of cases	Incomplete outcome data (attrition bias)	Score	Risk of bias
Qixin Zhang [18]	Random	Unclear	Unclear	Unclear	Low	Low	3	Unclear
Yinjuan Huang [19]	Random	Unclear	Unclear	Unclear	Low	Low	3	Unclear
Changfeng Zhang [20]	Random	Unclear	Unclear	Unclear	Low	Low	3	Unclear
Lianfen Bai [21]	Random	Unclear	Unclear	Unclear	Low	Low	3	Unclear
Lin Li [22]	Random	Unclear	Unclear	Unclear	Low	Low	3	Unclear
Haiying Lu [23]	Random	Unclear	Unclear	Unclear	Low	Low	3	Unclear
Cuilan Zhou [24]	Random	Unclear	Unclear	Unclear	Low	Low	3	Unclear
Ting Zhu [25]	Random	Unclear	Unclear	Unclear	Low	Low	3	Unclear
Huiqiang Yu [26]	Random	Unclear	Unclear	Unclear	Low	Low	3	Unclear
Xinyan Liu [27]	Random	Unclear	Unclear	Unclear	Low	Low	3	Unclear
Yan Jiang [28]	Random	Unclear	Unclear	Unclear	Low	Low	3	Unclear
Min Song [29]	Random	Unclear	Unclear	Unclear	Low	Low	3	Unclear
Cuilian Feng [30]	Random	Unclear	Unclear	Unclear	Low	Low	3	Unclear
Anxing Liu [31]	Random	Unclear	Unclear	Unclear	Low	Low	3	Unclear
Yueping Deng [32]	Random	Unclear	Unclear	Unclear	Low	Low	3	Unclear
Chunyan Zhang [33]	Random	Unclear	Unclear	Unclear	Low	Low	3	Unclear
Rong Li [34]	Random	Unclear	Unclear	Unclear	Low	Low	3	Unclear
Shizhen Zhong [35]	Random odd-even number	Unclear	Unclear	Unclear	Low	Low	3	Unclear
Fang Liu [36]	Random	Unclear	Unclear	Unclear	Low	Low	3	Unclear
Guihua Liu [37]	Random number table	Low	Unclear	Unclear	Low	Low	4	Unclear
Zhimin Xu [38]	Random number table	Low	Unclear	Unclear	Low	Low	4	Unclear
Rui Chen [39]	Random	Unclear	Unclear	Unclear	Low	Low	3	Unclear
Qiulian Deng [40]	Random	Unclear	Unclear	Unclear	Low	Low	3	Unclear
Xuezhen Zhang [41]	Random	Unclear	Unclear	Unclear	Low	High	2	High
Hong Chen [42]	Random number table	Low	Unclear	Unclear	Low	Low	4	Unclear

### 3.5. Meta-analysis result

#### 3.5.1. Cure rate

**Figure 4.**
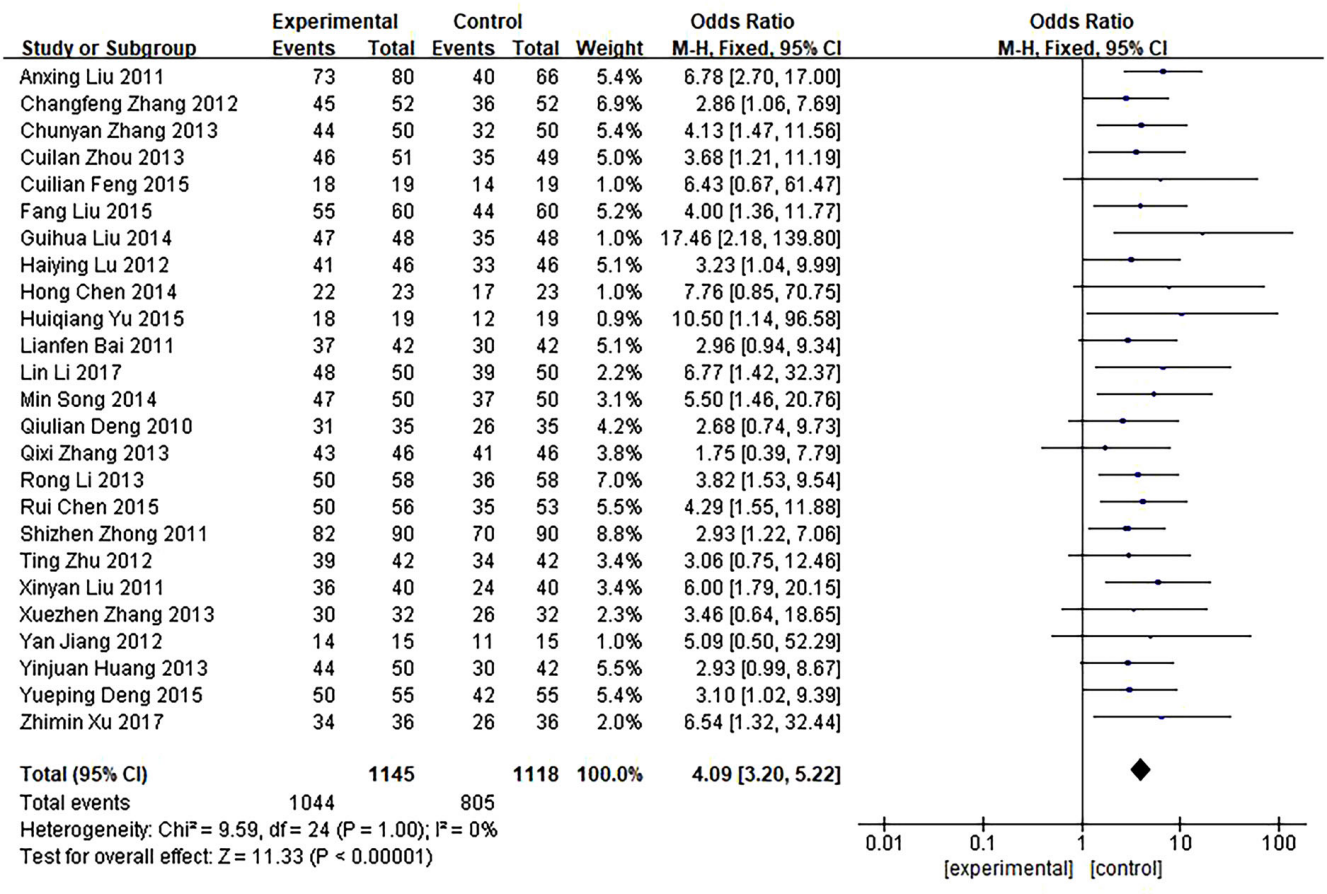
Forest plot of comparison: cure rate.

**Figure 5.**
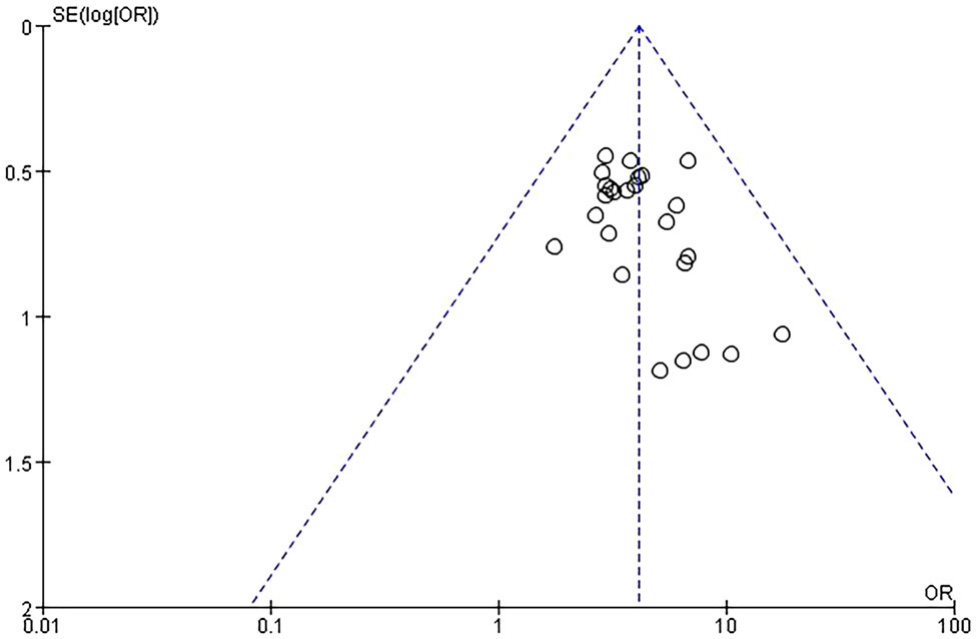
Funnel plot of comparison: cure rate.

Cure rate used as the primary outcome indicator in 25 trials involving a total of 2263 patients were included and showed a significant difference in clinical efficacy between the investigational groups and the control groups. *Q*-test and *I*² test were used, which showed low heterogeneity of the trial results (*χ*² = 9.59, *p* = 1.00, *I*² = 0%), therefore, a fixed-effects model (FE) was used for statistical analysis. Results showed that the cure rate of the investigational group was higher than that of the control group, indicating that the curative effect of the investigational group was better than the control group (*n* = 2263, OR = 4.09, 95%CI: [3.20, 5.22], *Z* = 11.33, *p* < 0.00001), and the difference was statistically significant. In addition, a funnel plot was obtained by publication bias analysis, funnel plot asymmetry showed potential publication bias for the absence of negative results or lack of large sample studies. Detailed results are shown in Figures 4 and 5.

#### 3.5.2. β-HCG level

**Figure 6.**
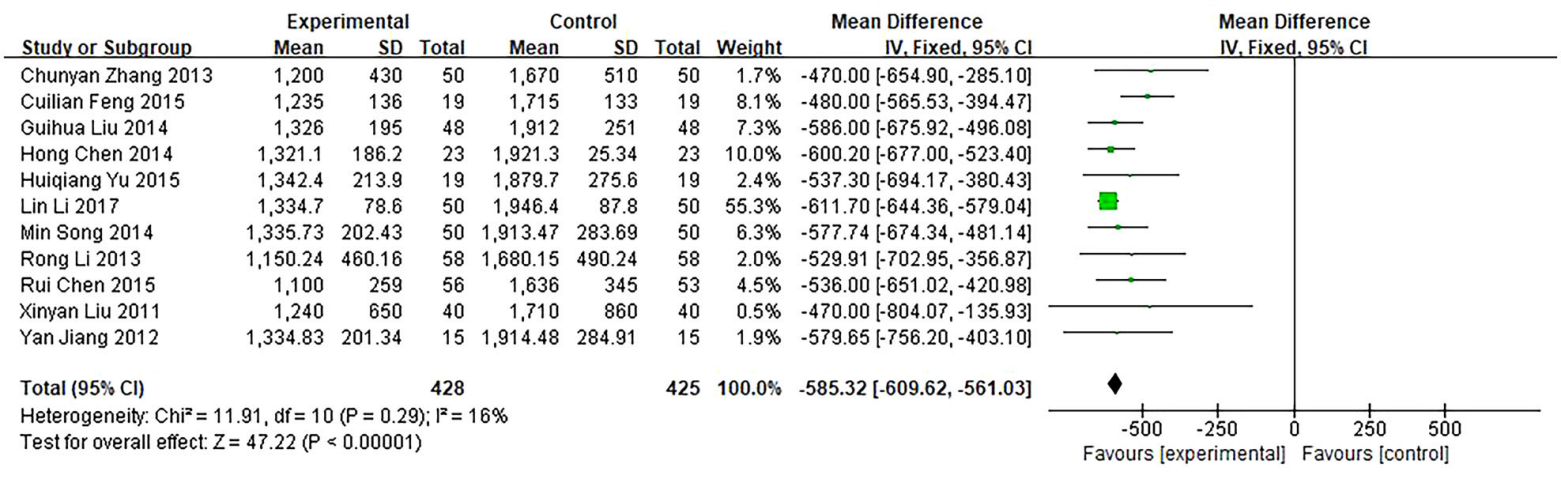
Forest plot of comparison: β-HCG Level.

**Figure 7.**
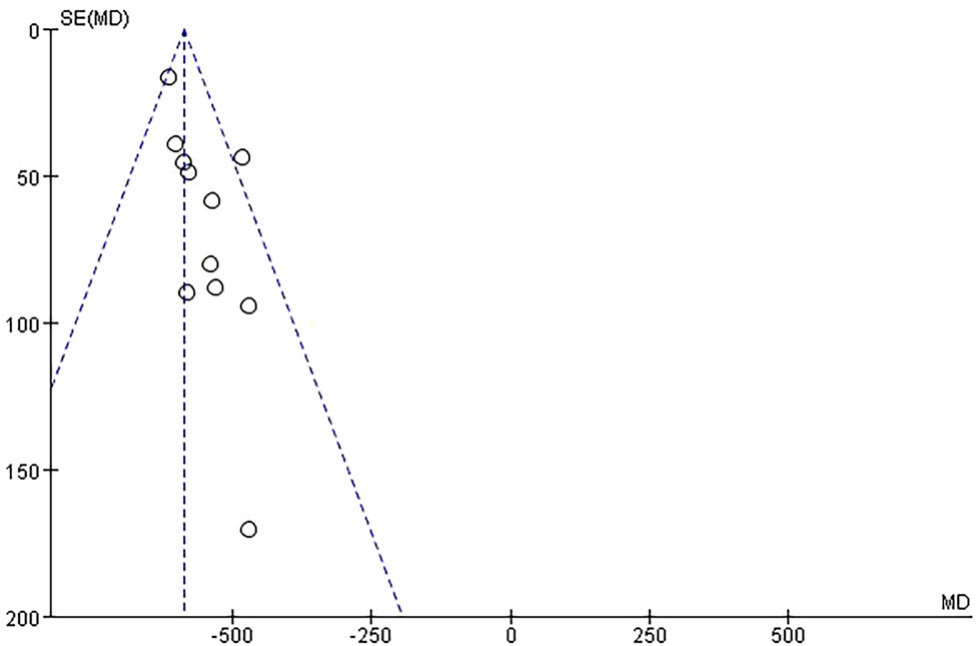
Funnel plot of comparison: β-HCG Level.

Thirteen trials reported β-HCG level as an outcome. The unit of the outcome in 2 trials [32,40] was inconsistent with that in the rest trials, so they were removed. The remaining 11 trials, involving a total of 853 patients, were included and showed a significant difference between the investigational groups and the control groups. First, *Q*-test and *I*² test were used, which showed low heterogeneity of the trial results (*χ*² = 11.91, *p* = 0.29, *I*² = 16%), therefore, a fixed-effects model (FE) was used for statistical analysis. Results showed that the β-HCG level of the investigational group was lower than the control group, indicating that the curative effect of the investigational group was better than the control group (*n* = 853, MD = −585.32, 95%CI: [−609.62, −561.03], *Z* = 47.22, *p* < 0.00001). and the difference was statistically significant. In addition, a funnel plot was obtained by publication bias analysis, funnel plot asymmetry showed potential publication bias for the absence of negative results or lack of large sample studies (Figures 6 and 7).

#### 3.5.3. Time of β-HCG returning to normal

**Figure 8.**
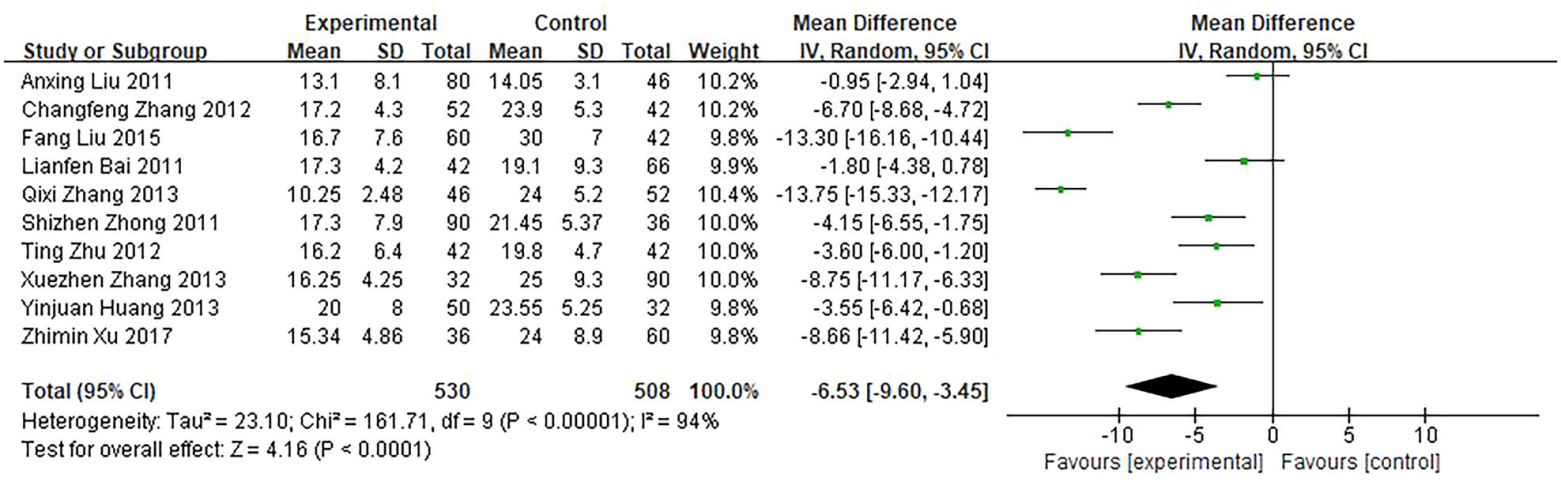
Forest plot of comparison: time of β-HCG returning to normal.

Ten trials, involving 1038 patients, reported time of β-HCG returned to normal as an outcome were included and showed a significant difference between the investigational groups and the control groups. *Q*-test and *I*² test were used, which showed high heterogeneity of the trial results (Tau² =  23.10, *χ*² =  161.71, *p* < 0.00001, *I*² =  94%), thus, a random-effects model (RE) was used for statistical analysis. The results showed that the time of β-HCG returned to normal in the investigational group was shorter than the control group, indicating that the curative effect of the investigational group was better than the control group (*n* = 1038, MD = −6.53, 95%CI: [−9.60, −3.45], *Z* = 4.16, *p* < 0.0001), and the difference was statistically significant (Figure 8).

#### 3.5.4. Time of vagina to stop bleeding

**Figure 9.**
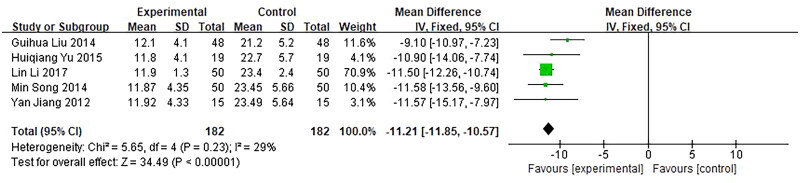
Forest plot of comparison: time of vagina to stop bleeding.

Five trials, involving 364 patients, reported the time of the vagina stopped bleeding as an outcome. They were included in our meta-analysis and showed a significant difference between the investigational groups and the control groups. *Q*-test and *I*² test was used, which showed low heterogeneity of the trial results (*χ*² =  5.65, *p* = 0.23, *I*² =  29%), therefore, a fixed effects model (FE) was used for statistical analysis. The results showed that the time of vagina stop bleeding of the investigational group was shorter than the control group, indicating that the curative effect of the investigational group was better than the control group (*n* = 364, MD = −11.21, 95%CI: [−11.85, −10.57], *Z* = 34.49, *p* < 0.00001), and the difference was statistically significant (Figure 9).

#### 3.5.5. Time of disappearance of abdominal pain

**Figure 10.**
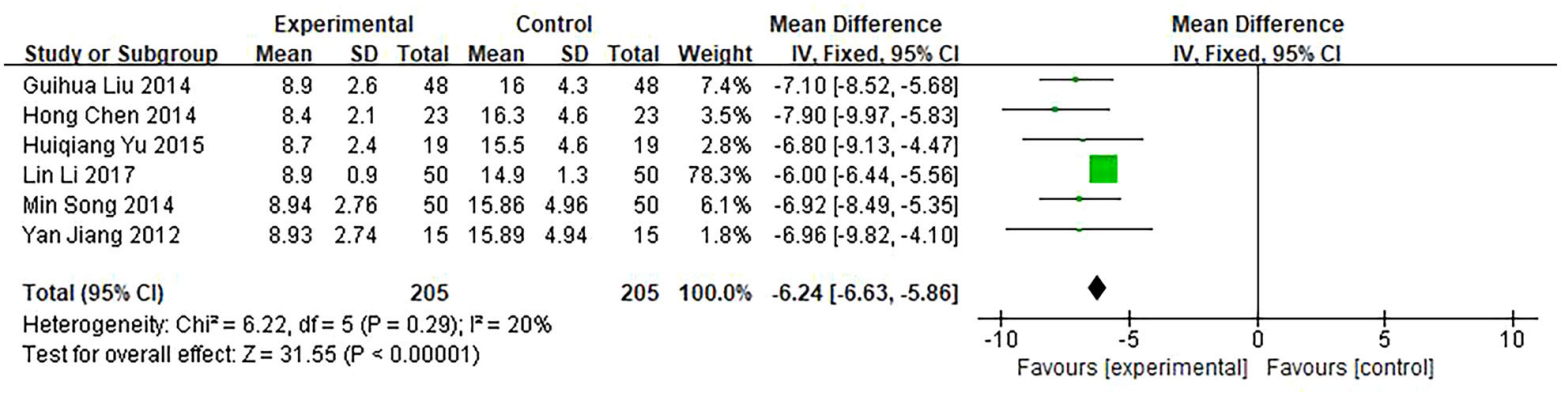
Forest plot of comparison: time of disappearance of abdominal pain.

Six trials, involving 410 patients, reported time of abdominal pain disappeared as an outcome. They were included in our meta-analysis and showed a significant difference in clinical efficacy between the investigational groups and the control groups. *Q*-test and *I*² test were used, which showed low heterogeneity of the trial results (*χ*² =  6.22, *p* = 0.29, *I*² =  20%), therefore, a fixed-effects model (FE) was used for statistical analysis. The results showed that the time of abdominal pain disappeared in the investigational group was shorter than in the control group, indicating that the curative effect of the investigational group was better than the control group (*n* = 410, MD = −6.24, 95%CI: [−6.63, −5.86], *Z* = 31.55, *p* < 0.00001), and the difference was statistically significant (Figure 10).

#### 3.5.6. Time of disappearance of the mass

**Figure 11.**
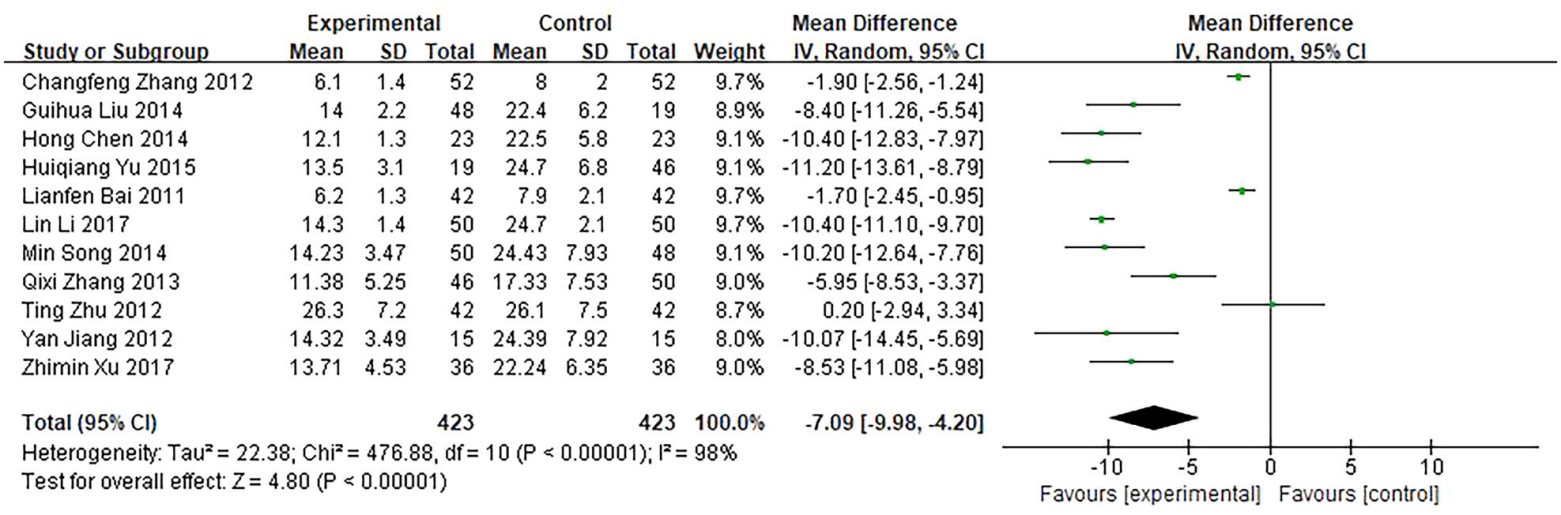
Forest plot of comparison: time of disappearance of the mass.

Eleven trials, involving 846 patients, reported the time of the mass disappearance as an outcome. They were included in our meta-analysis and showed a significant difference in clinical efficacy between the investigational groups and the control groups. *Q*-test and *I*² test were used, which showed high heterogeneity of the trial results (Tau² =  22.38, *χ*² =  476.88, *p* < 0.00001, *I*² =  98%), therefore, a random-effects model (RE) was used for statistical analysis. The results showed that the time of the mass disappearance of the investigational group was shorter than the control group, indicating that the curative effect of the investigational group was better than the control group (*n* = 846, MD = −7.09, 95%CI: [−9.98, −4.20], *Z* = 4.80, *p* < 0.00001), and the difference was statistically significant (Figure 11).

#### 3.5.7. Diameter of the mass

**Figure 12.**
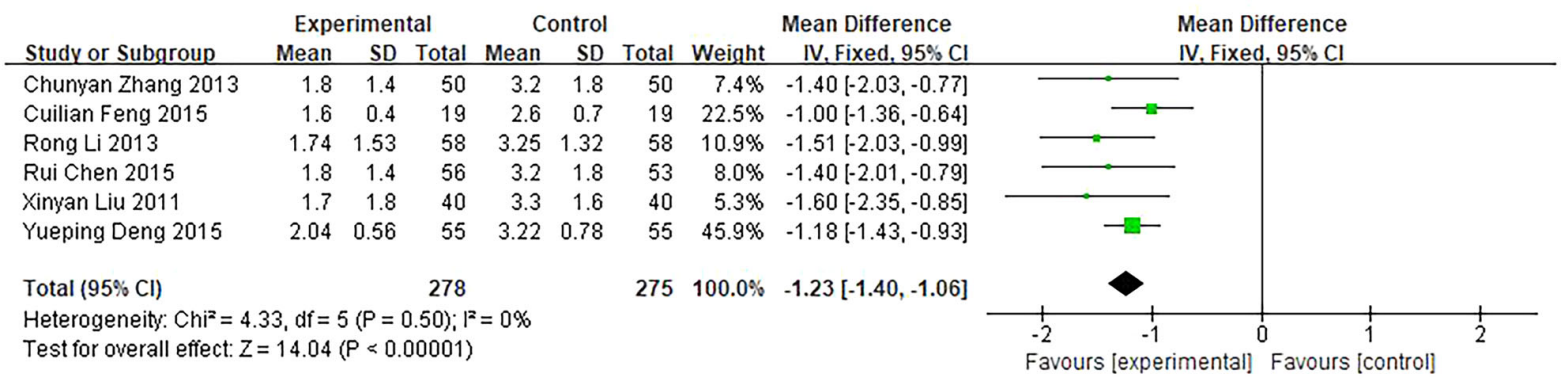
Forest plot of comparison: diameter of the mas.

Seven trials reported the diameter of the mass as an outcome. The diameter was recorded dynamically in 1 trial [40] was inconsistent with that in the rest trials, so it was removed. The remaining 6 trials, involving 553 patients, were included in our meta-analysis and showed a significant difference between the investigational groups and the control groups. *Q*-test and *I*² test were used, which showed low heterogeneity of the trial results (*χ*² =  4.33, *p* = 0.50, *I*² =  0%), thus, a fixed-effects model (FE) was used for statistical analysis. The results showed that the diameter of the mass of the investigational group was smaller than the control group, indicating that the curative effect of the investigational group was better than the control group (*n* = 553, MD = −1.23, 95%CI: [−1.40, −106], *Z* = 14.04, *p* < 0.00001), and the difference was statistically significant (Figure 12).

### 3.6. Certainty of evidence (GRADE)

Using the GRADE system recommendation approach, the certainty of the evidence for β-HCG level and diameter of the mass is high, as the certainty of the evidence for cure rate, time of vagina stopped bleeding, time of abdominal pain disappeared and time of the mass disappeared is moderate, the certainty of the evidence for the time of β-HCG returned to normal is low. The demotion is mainly due to the risk of bias (subjective symptom easily affected by unclear risk because of no blinding or high risk of bias ) and Inconsistency (significant statistical heterogeneity). Table 4 provides a summary of the certainty of available evidence.

**Table 4.** GRADE evaluation form of evidence certainty.

Patient or population: Ectopic Pregnancy Intervention: Combination of Mifepristone and Methotrexate Comparison: Mifepristone					
Outcomes	Anticipated absolute effects^A^ (95% CI)		Relative effect (95% CI)	№ of participants (studies)	Certainty of the evidence (GRADE)
	Risk with comparison	Risk with intervention			
Cure rate	720 per 1000	913 per 1000	OR 4.09	2263	⨁⨁⨁◯
		(892–931)	(3.20 to 5.22)	(25 RCTs)	Moderate^a^
β-HCG level		MD 585.32 lower	**-**	853	⨁⨁⨁⨁
		(609.62 lower to 561.03 lower)		(11 RCTs)	High
Time of β-HCG returned to normal		MD 6.53 lower	**-**	1038	⨁⨁◯◯
		(9.6 lower to 3.45 lower)		(10 RCTs)	Low^b,c^
Time of vagina stopped bleeding		MD 11.21 lower	**-**	364	⨁⨁⨁◯
		(11.85 lower to 10.57 lower)		(5 RCTs)	Moderatea^a^
Time of abdominal pain disappeared		MD 6.24 lower	**-**	410	⨁⨁⨁◯
		(6.63 lower to 5.86 lower)		(6 RCTs)	Moderatea^a^
Time of the mass disappeared		MD 7.09 lower	**-**	846	⨁⨁⨁◯
		(9.98 lower to 4.2 lower)		(11 RCTs)	Moderatea^b^
Diameter of the mass		MD 1.23 lower	**-**	553	⨁⨁⨁⨁
		(1.4 lower to 1.06 lower)		(6 RCTs)	High

^A^The risk in the intervention group (and its 95% confidence interval) is based on the assumed risk in the comparison group and the relative effect of the intervention (and its 95% CI).

^a^Risk of bias (subjective symptom easily affected by unclear risk because of no blinding); ^b^Inconsistency (significant statistical heterogeneity); ^c^Risk of bias (high risk of bias).

GRADE Working Group grades of evidence: High certainty: we are very confident that the true effect lies close to that of the estimate of the effect. Moderate certainty: we are moderately confident in the effect estimate: the true effect is likely to be close to the estimate of the effect, but there is a possibility that it is substantially different. Low certainty: our confidence in the effect estimate is limited: the true effect may be substantially different from the estimate of the effect. Very low certainty: we have very little confidence in the effect estimate: the true effect is likely to be substantially different from the estimate of effect.

## 4. Discussion

EP is a common acute abdominal disease in gynaecology. In recent years, due to increasing life pressure and unhealthy living habits, the incidence of EP is significantly more than that in the past and showed a younger trend. Considering most EP patients are young childless women, conservative medical treatment is chosen to preserve their fallopian tube function, so as to achieve the best treatment effect. Methotrexate is a folic acid antimetabolite, and it is currently recognized as the first choice for the treatment of uterine pregnancy. Mifepristone, a progesterone antagonist, can also kill embryos, however, there are certain limitations in the use of mifepristone alone. Since mifepristone and methotrexate work differently in ectopic pregnancy, does their combination really amplify the effect, reduce β-HCG levels, shrink the mass and relieve symptoms more efficiently? Will their combination amplify the toxic side effects? That is what we are going to talk about in more detail. Therefore, this study will systematically evaluate the efficacy of mifepristone combined with methotrexate therapy for EP, according to the meta-analysis of currently published clinical trials.

### 4.1. Analysis of efficacy

Twenty-five trials, involving 2263 patients, are included in this meta-analysis. The result shows that the combination of mifepristone and methotrexate therapy is better than the mifepristone alone therapy for EP. In addition, compared with the mifepristone alone therapy, the combination therapy can improve the cure rate to a greater extent, lower β-HCG level, minimize mass diameter and improve symptoms such as vaginal bleeding and abdominal pain more rapidly. Therefore, these results support the combination of mifepristone and methotrexate as a therapeutic strategy to improve the curative effect of conservative treatment for EP.

This meta-analysis used cure rates as the primary indicator, as shown in Figure 4, reliable and consistent results were produced to support the benefit of mifepristone and methotrexate combination therapy in improving the cure rate of EP. Although most of the trials had the same definition of the diagnostic criteria for the cure, there were still some trials with more detailed requirements on it. For example, in the trial of Qixin Zhang [18], the diagnostic criteria for the cure were divided as follows: symptoms disappear and blood β-HCG less than 0.88 μg/L for two consecutive times, gynecological examination and ultrasound examination show that the mass of EP in the pelvic cavity disappeared or the haematoma mass in pelvic cavity decreases by more than 50%.

Our meta-analysis results showed that mifepristone combined with methotrexate therapy could lower β-HCG levels significantly better than mifepristone alone therapy. In fact, although the 11 trials vary in certain aspects, including initial β-HCG levels, medication time and dose fractionated taking and so on, the heterogeneity is low and consistency is obvious. Therefore, the meta-analysis results support that mifepristone combined with methotrexate can more effectively reduce β-HCG levels. According to the principle of pharmaceutical effect, trophoblastic cells secrete a large number of HCG during pregnancy, and gestational trophoblastic cells have high sensitivity to methotrexate and they can not continue to grow and can be dissolved more quickly. Furthermore, mifepristone competitively binds to the progesterone receptor activation domain and antagonizes the progesterone activity. It can bind to the hormone-binding domain at the progesterone receptor(PR)-N tail due to the characteristics of its phenyl group at the 11β site, and it is 5 times stronger than PR affinity, which may lead to the result that mifepristone has better efficacy in the EP with high levels of progesterone. By reason of the foregoing, the combination therapy has a better curative effect to lower the HCG level.

Ten trials with the time of β-HCG returning to normal as the outcome indicator showed better efficacy in the investigational group, but there was significant heterogeneity. It may be due to the differences in the initial β-HCG level and dosage and treatment course and location between trials, which has a great influence on this outcome indicator. Thus, the time of β-HCG returning to normal as an outcome indicator is not quite effective in evaluating drug efficacy.

Five trials with the time the vagina stopped bleeding as the outcome indicator and 6 trials with the time of abdominal pain’s disappearance as the outcome indicator have low heterogeneity. As the EP progresses, the pregnancy sac gradually increases and the patient may experience sudden and severe abdominal pain, irregular vaginal bleeding and other symptoms. Studies have shown that the combination of mifepristone and methotrexate has a synergistic effect on EP, double blocking the embryonic development, mutually enhancing efficacy, effectively killing trophoblast cells and jointly promoting mass absorption, thereby accelerating the disappearance of vaginal bleeding and abdominal pain and other symptoms. The results of our meta-analysis bear this out.

Six trials with the diameter of the mass as the outcome indicator have no heterogeneity (*I*² =  0). It can be seen that the variable factors such as inconsistency of mass initial size, treatment course and measurement methods have little or even negligible influence on the diameter of the mass between trials. Therefore, it can be accurately and reliably concluded that the investigational group is more efficient than the control group in killing trophoblast cells, promoting mass absorption and reducing the diameter of the mass. Seven trials with the time of the disappearance of the mass as an outcome indicator showed that the observation group had better efficacy, but the heterogeneity was significant. It may be due to the differences in the initial mass diameter and dosage and treatment course and measurement methods between trials, which have a great influence on this outcome indicator. Thus, the time of the mass disappearance as an outcome indicator is not quite effective in evaluating drug efficacy.

The principle of methotrexate in treating EP is that it has a high affinity with dihydrofolate reductase and binds to it in a competitive manner. During the treatment, the growth of trophoblast cells is blocked and the placental villi are damaged, so that the embryo stops developing and the tissues are necrotic and fall off. Mifepristone is a kind of receptor-level antiprogesterone drug that competes with progesterone for receptors and terminates a pregnancy that depends on luteal maintenance. According to the results above, the combination of mifepristone and methotrexate in the treatment of EP can improve the cure rate more effectively, more significantly reduce β-HCG level, improve symptoms such as vaginal bleeding and abdominal pain and promote the absorption of mass. Thus, combination therapy has better clinical efficacy compared with mifepristone-alone therapy.

However, there were limitations in this meta-analysis. Among the included trials, some studies had different dosages and courses of treatment. For example, some trials used mifepristone 50 mg per time while some trials used mifepristone 150 mg on the first day and 75 mg per time on the 2nd and 3rd days. The definition of cure is also not entirely consistent in some trials, with some trials requiring a sustained weekly decline of more than 15% of β-HCG, and others requiring a decline to normal levels. There were also differences between the treatment courses, the treatment course was 5 days for some trials while 3 or 7 days for the others. Since no trials performed sample size calculations, it is difficult to assess clinical efficacy, which may negatively affect the reliability of the results.

Moreover, although mifepristone competes with progesterone to bind progesterone receptors, its affinity is 2–10 times of progesterone, but there are studies showing that the efficacy of mifepristone does not increase when progesterone level was >10 ng/L [43]. Thus, evaluation of pregnancy vascularisation is necessary because a higher vascularisation could suggest the presence of a wider syncytiotrophoblast and a consequent higher progesterone secretion. However, it was not taken into account in the included studies.

### 4.2. Quality of the evidence

Most randomized controlled trials were generally of poor quality in design, reporting and methodology, but good integrality in cases and outcome indicators, and good certainty of evidence. Random allocation was mentioned in all 25 trials, of which only 3 trials [37,38,42] clearly pointed out the use of the random number table. One trial [35] clearly pointed out the use of randomization based on odd and even numbers, while the rest of the trials only showed random allocation without detailed information. In all but three trials, allocation concealment was not mentioned, which may lead to selection bias. None of the trials mentioned the use of blinding. By the way, β-HCG level and mass diameter are objective indicators that are not easily affected by blinding, while subjective symptoms such as abdominal pain and vaginal bleeding are easily affected. Only one trial [41] lost a case in the follow-up of the tubal patency rate. However, only one trial used tubal patency rate as the outcome indicator, which was not included in the meta-analysis. Therefore, it could be considered that all the cases and outcome indicators were complete and there was no loss to follow-up bias. Moreover, except for the certainty of the evidence for the time of β-HCG returning to normal being low, other pieces of evidence have high or moderate certainty.

### 4.3. Potential biases

The funnel plots asymmetry demonstrates the potential publication bias. Due to no significant heterogeneity and the number of trials being more than 10, trials with cure rate and β-HCG level as indicators were included in these funnel plots. The funnel plots show that the distribution of trials is asymmetrical in the funnel plot, as there is a missing angle on the bottom left, suggesting publication bias or other bias, which may be due to the lack of large sample studies or the absence of negative results. This bias may affect the reliability of the meta-analysis.

### 4.4. Adverse event and safety

Adverse events were reported in most trials, and there was no significant tendency between the investigational groups and control groups. Eight trials did not mention the existence of adverse events, two trials specified no adverse events, one trial mentioned the existence of gastrointestinal reaction and no significant differences between the two groups, one trial showed the same incidence of adverse reactions in the two groups, four trials showed that the incidence of adverse reactions in the investigational group was higher than the control group, and nine trials showed that the incidence of adverse reactions in the control group was higher than the investigational group. Therefore, the combination therapy did not lead to more toxic side effects and had a certain safety level. Moreover, it is known that MTX toxicity may be distinct in different racial groups, the lack of ethnic diversity of participants in this study may limit the analysis of drug toxicity.

## 5. Conclusion

Our meta-analysis shows that compared with mifepristone therapy, the mifepristone combined with methotrexate therapy for EP can better improve the cure rate, and has a better curative effect on lowering β-HCG level, reducing the mass and alleviating symptoms of abdominal pain and bleeding. Moreover, combination therapy also has clear safety and no significant difference in the incidence of adverse reactions, which supports our conjecture that the combination of mifepristone and methotrexate can improve the efficacy of EP without amplifying the toxic side effects. However, most trials included were of poor quality, so the limitations of this meta-analysis should be taken into account. For further study, large-scale, well-designed and high-quality multicenter randomized controlled trials are needed to provide a better assessment. In addition, in order to evaluate the effect of the combination therapy on preserving the function of the fallopian tube and fertility, trials can also record the tube patency rate and repregnancy rate through long-term observation of prognosis.

Based on this meta-analysis, we suggest that the design of future trials should focus on the following aspects:

1. Improve the quality of trial design, including the evaluation of sample size, specific randomization, allocation concealment and blinding;
2. Scientific and consolidated standards and norms should be formulated, including the definition of cure, dosage and course of treatment;
3. Long-term prognostic effects of mifepristone combined with methotrexate therapy, such as fallopian tube patency rate and repregnancy rate, should be studied;
4. The site of onset of pregnancy can be taken into account and further studied.
5. The design of future trials should also take serum progesterone levels into account.

## Data Availability

The authors confirm that the data supporting the findings of this study are available within the article and its supplementary materials.

## References

[1] Guang G. Analysis of misdiagnosis of 24 EP cases. Jilin Med J. 2008;19:1670–1671.

[2] Jie L. Obstetrics and gynecology. 6th ed. Beijing (China): People’s Medical Publishing House, 2005. p. 110.

[3] Ying L. Analysis on the incidence trend and clinical diagnosis and treatment of EP. China Min-Kang Med. 2014;26(09):64–65.

[4] Lirong T, Ping Z. Decadal changes of EP risk factors. J Capital Med Univ. 2010;31(06):817–820.

[5] Naveed AK, Anjum MU, Hassan A, et al. Methotrexate versus expectant management in ectopic pregnancy: a meta-analysis. Arch Gynecol Obstet. 2022;305(3):547–553. 34524502 10.1007/s00404-021-06236-y

[6] Cuiling G. The value of quantitative detection of human chorionic gonadotropin in the early diagnosis of EP. J Shanxi Staff Med Coll. 2018;28(06):21–23.

[7] YuanYan Y. Observation and nursing care of 35 EP cases used conservative treatment. Qilu Nurs J. 2012;18(07):60–61.

[8] Wan S, Xiang Y, Fang W, et al. The effect of methotrexate in combination with mifepristone on EP: a meta-analysis. Int J Clin Exp Med. 2016;9(8):185.

[9] Insogna IG, Farland LV, Missmer SA, et al. Outpatient endometrial aspiration: an alternative to methotrexate for pregnancy of unknown location. Am J Obstet Gynecol. 2017;217(2):185.e1–185.e9. 28433735 10.1016/j.ajog.2017.04.023

[10] Bouchard P, Chabbert-Buffet N, Fauser BC. Selective progesterone receptor modulators in reproductive medicine: pharmacology, clinical efficacy, and safety. Fertil Steril. 2011;96(5):1175–1189. 21944187 10.1016/j.fertnstert.2011.08.021

[11] Xiao C, Shi Q, Cheng Q, et al. Non-surgical management of tubal ectopic pregnancy: a systematic review and meta-analysis. Medicine. 2021;100(50):e27851. 34918633 10.1097/MD.0000000000027851 PMC8677977

[12] Perdu M, Camus E, Rozenberg P, et al. Treating EP with the combination of mifepristone and methotrexate: a phase II nonrandomized study. Am J Obst Gynecol. 1998;179(3):640–643. 9757964 10.1016/s0002-9378(98)70057-2

[13] Xinlei C, Junna Z. Clinical observation on the treatment of EP with methotrexate, mifepristone, and their combination. China Mat Child Health Care. 2011;26(02):297–298.

[14] Heikinheimo O, Leminen A, Cacciatore B, et al. Advanced cervical pregnancy: uterus-sparing therapy initiated with a combination of methotrexate and mifepristone followed by evacuation and local hemostatic measures. Acta Obstet Gynecol Scand. 2004;83(2):211–213. 14756743 10.1111/j.0001-6349.2004.0077a.x

[15] Gómez García MT, Aguarón Benitez G, Barberá Belda B, et al. Medical therapy (methotrexate and mifepristone) alone or in combination with another type of therapy for the management of cervical or interstitial ectopic pregnancy. Eur J Obstet Gynecol Reprod Biol. 2012;165(1):77–81. 22771188 10.1016/j.ejogrb.2012.06.024

[16] Stabile G, Romano F, Buonomo F, et al. Conservative treatment of interstitial ectopic pregnancy with the combination of mifepristone and methotrexate: our experience and review of the literature. Biomed Res Int. 2020;2020:8703496. 32802882 10.1155/2020/8703496 PMC7421079

[17] Rozenberg P, Chevret S, Camus E, et al. Medical treatment of ectopic pregnancies: a randomized clinical trial comparing methotrexate-mifepristone and methotrexate-placebo. Hum Reprod. 2003;18(9):1802–1808. 12923131 10.1093/humrep/deg344

[18] Qixin Z, Fengqun W, Weijian D. Clinical observation of conservative treatment of EP. Contemporary Med. 2013;19(06):93–94.

[19] Yinjuan H, Junning S. Efficacy of methotrexate combined with mifepristone in the treatment of EP. World Latest Med Info Dig. 2013;9:207–207.

[20] Changfeng Z. Clinical efficacy of methotrexate combined with mifepristone in the conservative treatment of EP of 52 cases. Contemporary Med. 2012;18(30):58–59.

[21] Lianfen B, Baohuan Z, Limei S, et al. Therapeutic effect of methotrexate combined with mifepristone in the conservative treatment of EP. Chin Women Child Health. 2011;19(06):208.

[22] Lin L. Clinical efficacy of methotrexate combined with mifepristone in conservative treatment of EP. World Latest Med Info Dig. 2017;98:68.

[23] Haiying L. Clinical efficacy of methotrexate combined with mifepristone in the conservative treatment of EP. Chin J Med Guide. 2012;14(11):1934–1936.

[24] Cuilan Z. Clinical observation of methotrexate combined with mifepristone in non-operative treatment of EP. Chin J Clin Rational Drug Use. 2013;19:81–82.

[25] Ting Z. Clinical analysis of methotrexate combined with mifepristone in EP. Strait Pharma J. 2012;24(09):108–109.

[26] Huiqiang Y. Efficacy analysis of methotrexate combined with mifepristone in the treatment of EP. Chin J Modern Drug Appl. 2016;10(5):134–135.

[27] Xinyan L. Efficacy of methotrexate combined with mifepristone in the treatment of EP. China Pharma. 2011;22(20):1874–1876.

[28] Yan J. Clinical observation of methotrexate combined with mifepristone in the treatment of EP. Chin J Trauma Disabil Med. 2012;20(08):66–67.

[29] Min S. Clinical evaluation of methotrexate combined with mifepristone in the treatment of EP. Contemporary Med. 2014;20(24):33–34.

[30] Cuilian F. Clinical analysis of methotrexate combined with mifepristone in the treatment of EP. Contemporary Med. 2015;21(04):125–126.

[31] Anxing L. The clinical application of methotrexate combined with mifepristone in the treatment of tubal pregnancy. Med Innov China. 2011;8(2):56–57.

[32] Yueping D. Methotrexate combined with mifepristone in the treatment of tubal pregnancy. Med Info. 2015;28(39):233–234.

[33] Chunyan Z. Methotrexate combined with mifepristone in the treatment of 50 cases of EP. China Pharma. 2013;22(01):74–75.

[34] Rong L, Xiaoqin Y. Clinical analysis of methotrexate combined with mifepristone in the treatment of 58 cases of EP. China Health Care Nutr. 2014;24(2):991.

[35] Shizhen Z. Clinical observation of 90 cases of EP treated with methotrexate combined with mifepristone. Guangxi Med J. 2011;33(07):845–846.

[36] Fang L. Clinical observation of methotrexate combined with mifepristone in the treatment of 120 cases of EP. Capital Food Med. 2015;22(16):78.

[37] Guihua L. Efficacy of methotrexate combined with mifepristone in the treatment of EP. Chin J Clin Rational Drug Use. 2014;7(12):68–72.

[38] Zhimin X, Zhiling F. Clinical effect of methotrexate combined with mifepristone in treatment of EP. Jilin Med J. 2017;38(02):333–334.

[39] Rui C, Hua H, Shuyun M, et al. Treatment of EP with methotrexate combined with mifepristone. Chin J Woman Child Health Res. 2015;26(01):83–85.

[40] Qiulian D, Fengzhen L. Effect of methotrexate in treatment of EP. Contemporary Med. 2010;16(31):77–78.

[41] Xuezhen Z, Lanjuan F. Conservative treatment of EP with mifepristone combined with methotrexate in 64 patients. China Pharma. 2013;22(01):57–58.

[42] Hong C, Yan D. Effect of methotrexate combined with mifepristone in the treatment of EP. Contemporary Med Forum. 2014;12(17):156–157.

[43] Stabile G, Romano F, Zinicola G, et al. Interstitial ectopic pregnancy: the role of mifepristone in the medical treatment. Int J Env Res Public Health. 2021;18(18):9781. 34574706 10.3390/ijerph18189781 PMC8472240

